# Prevalence of Binocular Vision Anomalies and Refractive Error Among High School Students in Southern Trinidad: A Cross-Sectional Study

**DOI:** 10.22599/bioj.475

**Published:** 2025-08-22

**Authors:** Ngozika Esther Ezinne, Vishal Rattan, Safiyyah Mohansingh

**Affiliations:** 1 Optometry Unit, Department of Clinical Surgical Sciences, University of the West Indies, Saint Augustine Campus, Trinidad and Tobago; 2 Bathurst Rural Clinical School, School of Medicine, Western Sydney University, Australia; 3 Translational Health Research Institute, School of Medicine, Western Sydney University, Australia

**Keywords:** Refractive error, myopia, binocular vision anomalies, convergence insufficiency, high school students, Trinidad and Tobago

## Abstract

**Aim:**

This study aims to assess the prevalence of binocular vision anomalies and refractive errors among secondary school students in Southern Trinidad.

**Methods:**

A cross-sectional school-based study was conducted in two schools in southern Trinidad. Participants were randomly selected using an online spinner wheel. Refractive errors and binocular vision anomalies were assessed using handheld autorefractor and prism cover test respectively. Data on demographic information, refractive errors, and binocular vision were collected. The collected data were entered into Microsoft Excel and subsequently analysed using the Statistical Package for Social Sciences (SPSS). Descriptive statistics were computed using Pearson’s Chi-Squared test to analyse categorical variables, while Spearman’s rank correlation coefficient was used to assess the relationship between variables, with a significance level set at p < 0.05.

**Results:**

The study included 95 students, with 49 males (51.6%) and 46 females (48.4%), aged 12–18 years. The prevalence of binocular vision anomalies was 13.7% (13/95), with convergence insufficiency being the most common anomaly at 6.2%. Refractive errors were observed in 64.2% (61/95). of the participants, with myopia being the predominant type, affecting 54.2% of students. Spearman’s rank correlation test revealed no statistically significant correlation between binocular vision anomalies and refractive errors.

**Conclusion:**

The study identified a prevalence of 13.7% for binocular vision anomalies and 64.2% for refractive errors among secondary school students in Southern Trinidad. There is a need for a more comprehensive screening of binocular vision anomalies and refractive error for high school children in Trinidad.

## Introduction

Binocular vision is a critical visual process that allows for the integration and coordination of visual stimuli from both eyes into a single, unified image. This process is essential for depth perception and provides a broad field of view, contributing to a range of everyday activities, from reading to navigating complex environments. The brain’s ability to fuse these inputs relies on the effective functioning of the vergence and accommodative systems, which are integral for maintaining proper eye alignment and focusing on objects at various distances. When these systems fail to function properly, it can lead to binocular vision anomalies, a category of dysfunctions that impair the coordination of the two eyes and disrupt optimal visual performance (Candy and Cormack, 2022).

Binocular vision anomalies are generally classified into strabismic (heterotropia) and non-strabismic types, which include phorias, accommodative, and vergence anomalies. Among these, the most commonly encountered binocular vision anomalies are accommodative dysfunctions, convergence insufficiency, and heterophoria (Sharma *et al.*, 2024; Shrestha and Kaiti, 2022). Accommodative dysfunction involves difficulty in adjusting focus between near and far distances, while convergence insufficiency refers to an inability to align the eyes properly when focusing on near objects, leading to double vision or discomfort (Saikia *et al.*, 2024). Heterophoria is a misalignment of the eyes that occurs when binocular vision is disrupted but can be compensated for under normal conditions (Benhaim-Sitbon *et al.*, 2022). These anomalies can significantly reduce visual efficiency and diminish stereoacuity, the ability to perceive depth and three-dimensional structure (Ancona *et al.*, 2014). The impact of binocular vision anomalies is not only visual but can also affect an individual’s ability to perform academic and occupational tasks, leading to reduced academic performance and social challenges, particularly in children.

Refractive errors occur when the eye’s optical system fails to focus light properly onto the retina, leading to blurred vision (Ezinne and Mashige, 2018; Rajabpour *et al.*, 2024). The primary types of refractive errors include myopia, hyperopia (farsightedness), and astigmatism (irregular corneal curvature) (Rajabpour *et al.*, 2024; Schiefer *et al.*, 2016). Myopia is the most prevalent form of refractive errors worldwide, affecting approximately 2.5 billion individuals (Liang *et al.*, 2025; Nouraeinejad, 2021). The prevalence of myopia has been increasing rapidly in recent decades, with studies indicating that the global prevalence ranges from 44.9% to 52.4% in various populations (Alghamdi *et al.*, 2021; Besufikad *et al.*, 2022). This surge has led some studies to describe myopia as a “global epidemic” (Dolgin, 2015; Grzybowski *et al.*, 2020). Hyperopia and astigmatism are also widespread, though they are less frequently studied in comparison to myopia (Hashemi *et al.*, 2017).

The prevalence of binocular vision anomalies and refractive errors shows considerable variability across different regions and populations. In countries such as Saudi Arabia, India, Ghana, and Ethiopia, the prevalence ranged from 34.3% to 75.6% report the prevalence of binocular vision anomalies to range from 34.3% to 75.6% (Alghamdi *et al.*, 2021; Atiya *et al.*, 2020; Darko-Takyi *et al.*, 2016; Mondal, 2020). Myopia, in particular, is a significant public health concern due to its increasing prevalence, which has been linked to both genetic and environmental factors such as prolonged near work and reduced outdoor activity (Dolgin, 2015; Grzybowski *et al.*, 2020; Olusanya *et al.*, 2019). Furthermore, uncorrected refractive errors can have profound effects on children’s visual and developmental outcomes, contributing to amblyopia (lazy eye), poor academic performance, impaired social interactions, and a lower overall quality of life (Olusanya *et al.*, 2019). Therefore, early detection and correction of these conditions are vital for ensuring optimal visual and developmental outcomes.

Despite the substantial global focus on myopia and binocular vision anomalies, there remains a significant gap in research within the Caribbean region, including Trinidad and Tobago. This lack of local data hampers the development of targeted intervention strategies for addressing these visual disorders, which could significantly improve the educational and social outcomes for affected students. Therefore, this study aims to address this research gap by investigating the prevalence of binocular vision anomalies and refractive errors among secondary school children in Trinidad and Tobago. A better understanding of the local prevalence of these conditions is essential for crafting effective public health initiatives and educational strategies to support affected students, improving their overall quality of life and academic achievement.

## Method

### Study design

The study employed a cross-sectional school-based design to assess the prevalence of binocular vision anomalies and refractive errors among secondary school students in Trinidad and Tobago. A school-based study design was selected for this research as it provides a practical and efficient means of accessing a large, well-defined population of children and adolescents within the specified age range (12–18 years). Schools offer a centralized setting where students of similar developmental stages can be screened systematically, ensuring consistency in data collection and reducing logistical challenges associated with community-based recruitment. Additionally, since many refractive errors and binocular vision anomalies tend to emerge during the school years—often affecting academic performance and daily activities—schools represent a relevant and impactful setting for early detection. Conducting prevalence studies within schools also helps identify undiagnosed vision problems among students who may not otherwise seek or have access to regular eye care. This approach not only supports the research objectives but also contributes to public health efforts by facilitating timely referral and intervention for vision issues that may hinder learning and quality of life.

### Study setting

The Study was conducted in Trinidad and Tobago, a country with a total of approximately 199 secondary schools and an enrolment of 96,829 high school students with attendance rate of 97% (CSO, 2021). Of this total, 80,491 students are enrolled in government-run schools, while 11,515 students attend private institutions (CSO, 2021). This broad setting provided a diverse sample representing both public and private educational institutions. There are 49 government funded secondary schools in South Trinidad and high school attendance rate is 97% (CSO, 2021).

### Sampling size and technique

The sample size for the study was determined using the RAOSOFT sample size calculator, which recommended a minimum of 220 students to achieve a 95% confidence interval (Raosoft, Inc., n.d.). A multistage sampling technique was employed for participant selection.

### Inclusion and exclusion criteria

Students aged 12 to 18 years, enrolled in the 2022/2023 academic year, and who provided informed consent, were eligible for inclusion in the study. Exclusion criteria included students with a history of ocular trauma, active ocular pathology, or previous ocular surgeries, as these conditions could potentially confound the results by affecting visual function and the prevalence of binocular vision anomalies and refractive errors. This selective inclusion and exclusion process ensured a more homogeneous sample, minimising external variables that might interfere with the study’s objectives.

### Data collection procedures

The data was collected from September to December 2022. A multistage sampling technique was employed for participant selection. In the first stage, simple random sampling was used to select two secondary schools in Southern Trinidad. Subsequently, systematic random sampling was used to select a total of 22 students from each class/form (1–5) using an online spinner wheel. This involves first assigning each student a unique number or name on the spinner and dividing the sections of the spinner based on the number of students in the class and labelling each section of the spinner with a number that corresponds to the students’ names. The spinner wheel is then spun, and the student whose number lands on the chosen section was selected to participate in the study. Spinner was spun multiple times until desired number from each class was obtained. A total of 110 students was enumerated from each school. All students who were selected from the spinner were given information documents and consent forms for their parents. Those that consent was obtained from were included in the study.

Objective refraction was measured using the handheld autorefractor (Retinomax K Plus 3), with myopia defined as a spherical equivalent greater than –0.50 dioptres (D), hyperopia as greater than +0.50 D, and astigmatism as greater than –0.25 D cylinder (Alghamdi *et al.*, 2021). Visual acuity (VA) was assessed using the LogMAR chart, which provides a precise measurement of visual clarity (Rickham, 1964). Binocular heterophoria was evaluated using the alternating and unilateral prism bar cover test at 6 meters (distance) and 40 cm (near). Basic esophoria was characterised by equal esophoric deviations greater than 1 prism dioptre (PD) at both distances, while basic exophoria was identified by exophoric deviations greater than 6 PD at near and 3 PD at distance (Momeni-Moghaddam *et al.*, 2014; Themes, 2021).

Stereoacuity was measured using the Titmus Stereo-Fly test, with reduced stereoacuity defined as greater than 100 seconds of arc (SAR) (Tiwari *et al.*, 2022). The amplitude of accommodation (AA) was measured using the Donder’s push-up method with a Royal Air Force (RAF) rule. Accommodative insufficiency was determined using Hofstetter’s age-amplitude formula (K., 1947). Near point of convergence (NPC) was measured with the RAF rule, and convergence insufficiency was defined as a break value greater than or equal to 10 cm (Alghamdi *et al.*, 2021).

### Ethical considerations

Ethical approval was secured from the University of the West Indies Campus Research and Ethics Committee (Ref: CREC-SA.1809/10/2022). Permission to conduct the study in the secondary school was obtained from the Ministry of Education and principals of selected schools. Information and reason to participate in the study were explained to the participants. Informed written consent was obtained from both parents and students prior to participation. The study adhered to the ethical principles outlined in the Declaration of Helsinki (Rickham, 1964), ensuring the protection and confidentiality of participant data.

### Data analysis

Data were entered into Microsoft Excel and subsequently imported into IBM Statistical Package for the Social Sciences (SPSS) version 24 for analysis. Descriptive statistics, including frequencies, percentages, means, and standard deviations, were used to summarise the data. Pearson’s Chi-Square test was employed to describe the distribution of categorical variables. Associations between variables were assessed using Spearman’s rho correlation coefficient. Statistical significance was set at p < 0.05.

## Results

### Demographic profile of participants

A total of 95 participants aged 12 to 18 years, with a mean (± SD) age of 15.05 ± 1.8 years gave their consent and participated in the study. Most participants were males (51.6%), and the age group of 12–15 years was the most prevalent, comprising 58.95% of the sample (Table 1).

**Table 1:** Demographic profile of participants.


VARIABLE	GROUP	FREQUENCY (n = 95)	PERCENTAGE (%)

Age	12–15 years	56	58.9

	16–18 years	39	41.1

	**Total**	**95**	**100**

Gender	Male	49	51.6

	Female	46	48.4

	**Total**	**95**	**100**

Aided VA	Better than 0.3	35	89.7

	0.3 or worst	4	10.3

	**Total**	**39**	**100**

Binocular vision anomalies	Yes	13	13.7

	No	82	86.3

	**Total**	**95**	**100**

Unaided VA (LogMAR)	Better than 0.3	45	80

	0.3 or worst	11	20

	**Total**	**56**	**100**

Refractive error	Yes	61	64.2

	No	34	35.8

	**Total**	**95**	**100**

Use of Spectacles	Yes	39	41.1

	No	56	58.9

	**Total**	**95**	**100**


### Prevalence and demographic distribution of binocular vision anomalies

The prevalence of binocular vision anomalies was 13.7% (13/95) and convergence insufficiency was the most prevalent type of binocular vision anomalies (6.2%, n = 6). Binocular vision anomalies were found to be more prevalent among males and 12–15 age group although they were not statistically significant (p > 0.05) (Table 2).

**Table 2:** Prevalence and distribution of binocular vision anomalies.


VARIABLES		GROUP	FREQUENCY (n)	PERCENTAGE (%)

Binocular vision anomalies		Yes	13	13.7

		No	82	86.3

		**Total**	**95**	**100**

Accommodative Insufficiency		Yes	4	30.7

		No	9	69.3

		**Total**	**13**	**100**

Convergence Insufficiency		Yes	6	46.1

		No	7	53.9

		**Total**	**13**	**100**

Stereoacuity		Reduced	3	23

		Normal	10	77

		**Total**	**13**	**100**

Basic Esophoria		Yes	1	7.7

		No	12	92.3

		**Total**	**13**	**100**

**DEMOGRAPHY**	**p-VALUE**			

Age 12–15 years	0.058	Yes	9	16.1

		No	47	83.9

		**Total**	**56**	**100**

Age 16–18 years	0.058	Yes	4	103

		No	35	89.7

		**Total**	**39**	**100**

Male	0.070	Yes	7	14.3

		No	42	85.7

		**Total**	**49**	**100**

Female	0.070	Yes	6	13

		No	40	87

		**Total**	**46**	**100**


### Prevalence and demographic distribution of refractive error

The prevalence of refractive error was 64.2% (61/95). The most prevalent type of refractive error was myopia (53.6%, n = 51). No significant associations were found between refractive error, age, and gender (all p-values > 0.05) (Table 3).

**Table 3:** Prevalence and demographic distribution of refractive error.


CATEGORY		FREQUENCY (n)	SAMPLE PERCENT (%)	AGE (p-VALUE)	GENDER (p-VALUE)

Refractive error	Yes	61	64.2	0.05	0.32

	No	34	35.8	0.36	0.24

	**Total**	**95**	**100**		

Myopia	Yes	51	53.6	0.35	0.22

	No	44	46.4		

	**Total**	**95**	**100**		

Hyperopia	Yes	10	10.5	0.15	0.24

	No	85	89.5		

	**Total**	**95**	**100**		

Astigmatism	Yes	44	46.3	0.286	0.226

	No	51	43.7		

	**Total**	**95**	**100**		


### Correlation between binocular vision anomalies and refractive error

No significant associations were found between binocular vision anomalies and refractive error (all p-values > 0.05) (Table 4).

**Table 4:** Correlation between binocular vision anomalies and refractive error.


VARIABLE 1	VARIABLE 2	CORRELATION COEFFICIENT (r)	p-VALUE

Binocular vision anomalies	Myopia	0.04	0.72

Binocular vision anomalies	Hyperopia	0.01	0.94

Binocular vision anomalies	Astigmatism	0.07	0.51


**Correlation is significant at the 0.01 level (2-tailed).

## Discussion

This study represents the first effort to assess the prevalence of binocular vision anomalies and refractive errors among secondary school students in Trinidad and Tobago. The recorded prevalence rates were 13% for binocular vision anomalies and 64% for refractive errors. Among the various types of refractive errors and binocular vision anomalies, myopia, and convergence insufficiency emerged as the most prevalent conditions. The observed prevalence of binocular vision anomalies in this study was lower than the 34.3% reported by Darko-Takyi *et al.*, (Darko-Takyi *et al.*, 2016) in Ghana, as well as the broader range of 46% to 75% found in other studies (Alghamdi *et al.*, 2021; Atiya *et al.*, 2020; Mondal, 2020). Discrepancies in prevalence rates of binocular vision anomalies and refractive errors across studies could be attributed to cultural, environmental, and methodological differences. For example, this study focused specifically on secondary school students, a group that may have distinct visual habits, such as varying amounts of time spent reading or engaging in near work, compared to students in other countries. In regions such as Asia, where there is an increased emphasis on near work, including extensive reading or the frequent use of technology, myopia rates tend to be much higher, contributing to the growing global “epidemic” of myopia (Grzybowski *et al.*, 2020). Additionally, environmental factors like outdoor activity exposure—shown to reduce the likelihood of developing myopia (Dolgin, 2015) may further influence these regional variations.

Among the various types of binocular vision anomalies, convergence insufficiency emerged as the most prevalent, affecting 46.1% of individuals diagnosed with binocular vision anomalies, followed by accommodative insufficiency. These findings are consistent with those reported by Atiya *et al.*, (2020) who also identified a high prevalence of convergence insufficiency among specific student populations. This underscores the need for targeted interventions to manage convergence-related visual disorders in students. However, both our study and that of Atiya *et al.*, (2020) did not assess whether the identified cases of convergence insufficiency were symptomatic. Further research is needed to evaluate the presence and severity of symptoms, which would allow for more meaningful comparisons and a better understanding of the clinical relevance of these findings.

In terms of demographic patterns, binocular vision anomalies were more commonly observed among males and students aged 12–15 years, although these differences were not statistically significant. Research on gender differences in binocular vision anomalies prevalence has yielded mixed results. For instance, Mondal *et al.*, (2020) reported a higher prevalence in females, while Darko-Takyi *et al.*, (2016) found the opposite. The discrepancies in gender-related prevalence could be attributed to variations in study design, including differences in sampling methods and diagnostic criteria. For example, this study employed a multistage sampling technique, incorporating both public and private schools, whereas other studies may have had more homogenous samples, such as only public schools or rural versus urban settings. Additionally, differences in the age ranges and specific inclusion/exclusion criteria of participants might also account for the variability in findings, as certain age groups may be more prone to vision anomalies.

The study also found that 64.6% of participants had ametropia, with myopia being the most prevalent refractive error. The high prevalence of myopia and binocular vision anomalies, particularly among adolescents, has important implications for students’ academic performance. Uncorrected refractive errors can lead to significant difficulties in reading, concentrating, and participating in classroom activities, while untreated binocular vision anomalies can cause discomfort, headaches, and difficulty with tasks requiring near and far focusing. These visual challenges can adversely affect academic achievement and social development, underscoring the importance of integrating vision care into educational policies and practices. The findings of this study are consistent with those of Alghamdi *et al.*, (2021) who reported a myopia prevalence of 44.9%, and Besufikad *et al.*, (2022) with a reported prevalence of 52.4%. However, other studies (Atiya *et al.*, 2020; Darko-Takyi *et al.*, 2016; Olusanya *et al.*, 2019) reported lower prevalence rates for myopia, ranging from 17.1% to 24%. Hyperopia was observed in 10.5% of participants, with prevalence estimates in the literature ranging from 3% to 19% (Besufikad *et al.*, 2022; Darko-Takyi *et al.*, 2016). Alghamdi *et al.*, findings of 8.2% are most comparable to our results (Alghamdi *et al.*, 2021). The prevalence of astigmatism in this study was found to be 46.3%, which is notably higher than the 11% reported by Atiya *et al.*, (2020) and Besufikad *et al.*, (2022). These variations in prevalence may reflect differences in sample characteristics or methodological approaches, and further investigation is warranted to better understand these regional differences.

Significant association of age with RE found in our study aligns with findings from other studies (Ezinne and Mashige, 2018; Ma *et al.*, 2016; Santiago *et al.*, 2023). The association between age and refractive errors observed in this study can be attributed to a range of physiological and lifestyle-related factors that influence the eye across the lifespan. In early childhood, the eye undergoes significant growth and structural development, during which many individuals transition from the hyperopia typical of infancy to emmetropia or, in some cases, myopia—a process known as emmetropisation. However, gender did not significantly correlate with refractive error in this study. These findings contrast with studies by Olusanya *et al.*, (2019). who reported a higher prevalence of refractive error in males, and Besufikad *et al.*, (2022; Olusanya *et al.*, 2019) who found that females were four times more likely to have refractive errors than males. Furthermore, no statistically significant relationship was observed between accommodation insufficiency, convergence insufficiency, basic esophoria and refractive error, suggesting that the presence of binocular vision anomalies is not strongly associated with specific types of refractive error. These results are consistent with the findings of Tiwari *et al.*, (2022), Alghamdi *et al.*, (2021), and Atiya *et al.*, (2020), who also found no clear association between binocular vision anomalies and refractive errors. This may suggest that the underlying mechanisms of binocular vision anomalies and refractive errors are independent, warranting further exploration into the factors contributing to these conditions.

Spectacle utilisation rate (41.1%) recorded in our study was higher than 20.6% and 35.8% reported among school children in studies in Nigeria (Ezinne *et al.*, 2020) and China (Qian *et al.*, 2018). However, it was lower than 93% and 59.3% recorded in studies in India and Nepal respectively (Khatri *et al.*, 2024). These discrepancies in spectacle utilisation rates across different countries and regions may be influenced by several factors, including ethnic and racial differences, the characteristics of the study population, socio-economic status, accessibility, and affordability of eye care services, cultural attitudes toward spectacle use, and the type and severity of refractive errors common in each setting. Parental awareness, beliefs, and attitudes toward eye health also play a critical role in whether children consistently use their prescribed spectacles. The relatively low utilisation rates in some regions, despite the availability of spectacles, highlight a significant public health concern. Uncorrected refractive errors can impair academic performance, reduce quality of life, and hinder overall development in school-aged children. Therefore, it is essential to identify and address the barriers to spectacle use.

### Implications and recommendations for future studies

This study reveals a high prevalence of binocular vision anomalies and refractive errors among secondary school students in Trinidad and Tobago, with myopia and convergence insufficiency emerging as the most common conditions. These findings highlight the urgent need for integrating vision care into school health programs through regular, comprehensive vision screenings aimed at early detection and treatment. To address these challenges, public health campaigns should be developed to raise awareness among students, parents, and teachers about the importance of routine eye examinations. Enhancing access to affordable eye care services—particularly in underserved areas—is also essential to reduce the burden of uncorrected visual problems.

Schools should adopt standardised vision screening protocols and provide training for teachers and staff to identify and refer students with potential vision issues. Furthermore, revising school curricula to incorporate measures that reduce visual strain may contribute to lowering the risk of developing vision-related conditions such as myopia. Improving spectacle utilisation among school children is another critical priority. Targeted health education initiatives can help change perceptions and improve compliance, while school-based vision screening programs offering free or subsidised spectacles may increase uptake, particularly in low-resource settings. Additional research is needed to understand the barriers to spectacle use, including cultural, psychological, and systemic factors. Insights from such studies will be crucial for developing context-specific and sustainable interventions.

Future research should also aim to include larger, more diverse populations to improve generalisability. Longitudinal studies and intervention trials are recommended to better understand the natural history and progression of binocular vision anomalies and refractive errors, as well as to identify the most effective treatment and prevention strategies. Exploring both environmental and genetic determinants of visual health could further enhance our understanding of these conditions. Overall, this study underscores the need for a more integrated and proactive approach to vision care within educational systems and calls for evidence-based policies that prioritise eye health as a fundamental component of student well-being and academic success.

### Strengths and limitations

The study presents several important limitations that affect the interpretation and generalisability of its findings. The most notable limitation is the small sample size, which was constrained by COVID-19 restrictions. This directly impacts the statistical power of the study and reduces its ability to generalise findings to the broader population in Trinidad and Tobago. Additionally, the use of the Retinomax K-Plus 3 device, for assessing refractive error, introduces potential measurement bias. This tool is known to differ in its spherical power readings compared to the more traditional retinoscope, which may have affected the accuracy of the refractive assessments. Another limitation is the absence of binocular vision anomalies questionnaires. These tools could have helped in identifying symptomatic individuals or other forms of binocular vision anomalies that may not be detectable through standard testing alone. The lack of this information may result in underreporting or misclassification of certain conditions.

Despite these limitations, the study is significant as it is the first of its kind in Trinidad and Tobago. It lays important groundwork for future research and offers initial insights into the prevalence of binocular vision anomalies in the region.

## Conclusion

This study represents the first assessment of binocular vision anomalies and refractive errors prevalence among secondary school students in Trinidad and Tobago, and regionally in Latin America and the Caribbean. The prevalence of binocular vision anomalies was 13.7% (13/95 students), with convergence insufficiency being the most common type (6.2%, n = 6). Binocular vision anomalies were more prevalent among males and the 12–15 age group, though these differences were not statistically significant. The prevalence of refractive errors was 64.6% (51/95), with myopia being the most common refractive error (53.6%, n = 51). No significant associations were found between refractive errors and age or gender. The findings suggest that the prevalence of binocular vision anomalies and refractive errors in Trinidad and Tobago may be higher than reported, possibly due to the limited sample size. Regular school vision screening program is highly advised to enhance early detection and management. Future large population-based study that will assess the prevalence of refractive errors, binocular vision anomalies and utilisation of spectacles would be useful to understand the accurate prevalence in this region.

## Data Accessibility Statement

The data used in the study are available from the corresponding author upon a reasonable request.
